# Phenotypic plasticity in specialists: How long-spined larval *Sympetrum depressiusculum* (Odonata: Libellulidae) responds to combined predator cues

**DOI:** 10.1371/journal.pone.0201406

**Published:** 2018-08-08

**Authors:** Hana Šigutová, Martin Šigut, Aleš Dolný

**Affiliations:** 1 Institute of Environmental Technologies, Faculty of Science, University of Ostrava, Ostrava, Czech Republic; 2 Department of Biology and Ecology, Faculty of Science, University of Ostrava, Ostrava, Czech Republic; Department of Agriculture and Water Resources, AUSTRALIA

## Abstract

Phenotypic plasticity is a common defensive strategy in species experiencing variable predation risk, such as habitat generalists. Larvae of generalist dragonflies can elongate their abdominal spines in environments with fish, but long spines render larvae susceptible to invertebrate predators. Long-spined specialists adapted to fish-heavy habitats are not expected to have phenotypic plasticity in this defence trait, but no empirical studies have been undertaken. Moreover, in comparison to prey responding to multiple predators that induce similar phenotypes, relatively little is known regarding how species react to combinations of predators that favour opposing traits. We examined plasticity of larval dragonfly *Sympetrum depressiusculum*, a long-spined habitat specialist. In a rearing experiment, larvae were exposed to four environments: (i) no predator control, (ii) fish cues (*Carassius auratus*), (iii) invertebrate cues (*Anax imperator*), as well as (iv) a combination of (ii) and (iii). Compared with the control, fish but not invertebrate cues resulted in longer spines for two (one lateral, one dorsal) of the six spines measured. Interestingly, the combined-cue treatment led to the elongation of all four dorsal spines compared with the fish treatment alone, whereas lateral spines showed no response. Our experiment provided evidence of morphological plasticity in a long-spined specialist dragonfly. We showed that nearly all spines can elongate, but also react differently under specific predator settings. Therefore, while spine plasticity evolved in direct response to a single predator type (fish), plasticity was maintained against invertebrate predators as long as fish were also present. Selective spine induction under the combined condition suggests that *S*. *depressiusculum* can successfully survive in environments with both predators. Therefore, phenotypic plasticity may be an effective strategy for habitat generalists and specialists. Although more studies are necessary to fully understand how selection shapes the evolution of phenotypic plasticity, we demonstrated that in dragonflies, presence or absence of a specific predator is not the only factor that determines plastic defence responses.

## Introduction

Predation is a major selective agent of freshwater community structure, affecting prey phenotypes and population-level grouping patterns [1,2]. Prey have evolved numerous strategies to avoid predation, including modifying foraging behaviour, life history, and morphology (i.e. developing defensive structures [3–7]). The intensity of predator-prey interactions within natural food webs is relatively stable over evolutionary time but often exhibits considerable short-term spatiotemporal variation [8,9]. Thus, while permanent (constitutive) defences may be favoured in a constant environment, frequent unpredictability and the relative expense of constitutive morphological defences has caused many aquatic species to evolve inducible anti-predator responses [10–12]. Inducible defensive mechanisms are elicited only upon perceiving predator-specific water-borne chemical cues (‘kairomones’) [13] (but see [14,15] for details on terminology). This strategy is energy-efficient because unnecessary effort is not expended when predators are absent [16–18]. Indeed, the intensity of antipredator response is frequently correlated with predator-cue concentration [19,20].

Inducible defences are also favoured in organisms with complex life cycles, such as insects possessing aquatic larval stages. Furthermore, freely moving imago can oviposit into sites with variable predation risk, thus exposing subsequent generations to alternating selection pressure (reviewed by [9]). In the short term, phenotypic plasticity can extend a species’ habitat range, while also altering predator-prey populations and community dynamics [21]. Over evolutionary time, it can cause genetic differentiation and speciation [10,12,22].

Inducible defence mechanisms manifesting as behavioural, morphological, or life-history modifications have been reported for numerous freshwater taxa, including crustaceans, amphibians, and fish (e.g. [7,23–31]). Among aquatic insects or those with an aquatic life stage, mayfly larvae are known to develop defensive morphological characters in response to fish predators [32], while in mosquitoes and chironomids, fish cues suppress larval growth, leading to a life-history shift [33,34].

The ecology and evolution of inducible defences have been thoroughly studied in dragonflies of the genus *Leucorrhinia*. Larvae develop dorsal and lateral spines on the abdomen that provide protection against fish predators [35–37]. Spine length was confirmed to be plastic in both the laboratory and the field [35,38–41]. However, elongated spines facilitate grasping by hooks and palpi of the labium of large predatory dragonflies [42], which are typically top predators in lakes and ponds lacking fish [43]. Thus, fish and invertebrate predators exert antagonistic selection on spines as a defence mechanism [42]. Additionally, fish predation has been implicated in phenotypic diversification (and subsequently speciation) of *Leucorrhinia*, as larvae lost spines when shifting from fish-heavy to fishless habitats [41]. Therefore, morphological defensive traits can be linked to the preferred habitats and behavioural responses (e.g. escape) of *Leucorrhinia* to different predators [37,44,45].

Although the *Leucorrhinia* system is fairly well studied, we still do not know the answers to several important questions concerning their inducible morphological defences. Rearing experiments with fish predators confirmed phenotypic plasticity in several habitat generalists with intermediate spine length [39,41]: *L*. *dubia* [38,46] and *L*. *intacta* [39]. In contrast, a comparison of individuals from fish-heavy and fishless habitats suggests that long-spined species specializing in fish-heavy habitats did not alter spine length under fish exposure [41]. Thus, these long-spined larvae are thought to have constitutive spine expression, given that the advantages of having spines should far outweigh the disadvantages in a fish-heavy habitat [42]. However, this hypothesis has not been empirically tested with induction experiments among long-spined species, especially among non-*Leucorrhinia* species. Furthermore, previous induction experiments in dragonflies used fish predators only, assuming that the non-induced phenotype was a ‘default setting’ (short spines) that would be expressed against invertebrate predators. However, several studies (e.g. [47–50]) have revealed that prey can discriminate between predators and produce predator-specific phenotypes.

Here, we selected *Sympetrum depressiusculum* (Odonata: Libellulidae) as a representative long-spined dragonfly species that is well adapted to fish-heavy habitats. The species is a habitat specialist with restricted distribution [51–54]. We performed a rearing laboratory experiment that exposed larvae to continually released cues from fish or invertebrate predators (see [14]). Specifically, we tested two mutually exclusive hypotheses. First, the fixed spine length hypothesis suggests that fish-habitat specialists constitutively express long spines. Thus, we expect no spine-length differences between larvae reared without predator cues and larvae exposed to predator cues of any kind. Second, the plastic spine length hypothesis suggests that spine length is inducible, because *S*. *depressiusculum* adults disperse within a relatively wide home range [55] that encompasses different habitat types. Moreover, adults tend to have poor ability in selecting appropriate habitats for larval development [56]. Under this hypothesis, we expect spine elongation in larvae exposed to fish cues, but not when exposed to invertebrate cues, leading to a ‘default’ short spine length. In larvae exposed to both fish and invertebrate cues, we expect a dosage effect (i.e. diluted fish cues) that leads to an intermediate phenotype, as observed in other freshwater taxa (e.g. [19,20,23]).

Our study has the important distinction of controlling for diet cues (i.e. those released following the consumption of prey conspecifics [12]). Doing so teases apart the influence of different cues so that those stemming specifically from the predator (i.e. odour) should explain all observed effects. Such an experimental set-up is necessary for a clearer understanding of the mechanisms controlling antipredator responses (see [15]). Our results should provide new insight into inducible defensive morphological structures of prey facing multiple predators. More generally, our findings can help further current knowledge of factors driving the evolution of phenotypic plasticity.

## Material and methods

### Ethics statement

No specific permits were required for field sampling, as the sampled locality is not protected. The sampling locality is owned by the Czech Fishing Union, and the data were collected with their approval. No specific permissions were required to collect insect specimens, because the target species is not protected in the Czech Republic.

### Study organism

*Sympetrum depressiusculum* (Sélys, 1841) is a univoltine species (hibernating at the egg stage) ranging from Siberia to Western Europe. In the European red list it is ranked as vulnerable [52]. In Central Europe, larvae hatch in May (depending on the flooding of the habitat), and they usually have nine instars. Larval development lasts 6–8 weeks. Imagines emerge from July to mid-August and persist until early October [51,54]. Compared with other *Sympetrum* species, *S*. *depressiusculum* larvae possess very long lateral and dorsal spines on abdominal segments [57]. Their natural habitats are mainly pools and waterlogged meadows in the alluvial areas of rivers and lakes [51,58]; therefore, they commonly develop in habitats with fish as the top predators.

### Sample collection

During 10–25 August 2015, 12 mated pairs of *S*. *depressiusculum* were caught at a small pond used for rearing cyprinid phytophagous fish (*Chondrostoma nasus*), located near Příbor, Moravian-Silesian Region, Czech Republic (49.6347878N, 18.1012517E). Eggs were collected through trailing the tip of female abdomen through aged tap water (held for 24 hours before use) in a 200-mL transparent plastic container (diameter 5 cm, height 8.5 cm). This method prevented eggs from being exposed to predator cues before the experiment [39]. Clutches per female were placed separately into individual plastic containers filled with aged tap water, labelled with the female identification code, and transferred to the laboratory where they were stored at 5 °C.

### Induction experiment

We used predator-conditioned water (fish, invertebrate, and mixed treatment) and clean water (control), prepared as follows. Fish predators were represented by three 15–20 cm long *Carassius auratus* (Cyprinidae), an omnivorous predator of dragonfly larvae typically found in the temporary alluvial habitats of *S*. *depressiusculum* [59]. Fish were kept at 22 °C in a single 15-L aquarium filled with clean, aged tap water, without vegetation, and fed once a day a common fish-flake food. The tank was not aerated to avoid inhibiting kairomone activity [60].

Invertebrate predators were 25 ultimate-instar *Anax imperator* (Odonata: Aeshnidae) larvae sampled from a small fish breeding pond (same as used for *S*. *depressiusculum* sampling). These larval aeshnids are typically top predators of fishless environments [43,61] and commonly occur in *S*. *depressiusculum* habitats [54]. As part of a separate study, predatory larvae were kept in the laboratory for a year before the experiment. Maintenance conditions involved being in a single 15-L aquarium filled with non-aerated, clean, aged tap water (22 °C), as well as stones and sticks for roosting. Additionally, larvae were kept in the dark to avoid cannibalism and fed a live *Tubifex tubifex* from laboratory cultures every other day. Before each feeding, aquarium water was replaced with clean, aged tap water of the same temperature. During this procedure and two hours after, during feeding, the aquarium was lit, as *Anax* larvae detect prey visually [62].

For the mixed treatment (combined fish-invertebrate cues), we used 1:1 ratio of fish- and invertebrate-conditioned water, freshly obtained (and filtered) from predator aquaria. The control involved aged tap water that was kept in the same environmental conditions as predator-conditioned water, with the obvious exception of housing no predators. All aquaria (fish, invertebrate, control) were placed in the same room, and the water was synchronously replaced with clean, aged tap water of the same temperature every other day (before feeding *A*. *imperator* larvae). Removed water containing continually released predator-borne cues was then filtered through a soft cloth to remove dirt and used for the induction experiment.

The experiment began in January 2016. Containers with *S*. *depressiusculum* clutches were housed at 22 °C and a 14/10 h day/night cycle, to activate hatching. All containers were checked daily under a stereomicroscope, and newly hatched prolarvae were moved with a plastic dropper into new, same-sized plastic containers filled with predator-conditioned or control water, eventually forming four treatments (fish, invertebrate, mixed, control) each containing the same number of prolarvae. Once each morning and evening, prolarvae were fed live *Artemia salina* nauplii from laboratory cultures. Containers housing *S*. *depressiusculum* larvae were placed onto white LED strips for feeding, to ensure that the positively phototactic nauplii would drift downward where larvae were present. Because bacterial biodegradation gradually inactivates kairomones [60], water was changed post-evening feeding every other day for all treatments (including control). Containers were drained of water while the larvae remained inside, then refilled with fresh experimental or control water after food remains and faeces were removed with a plastic dropper. By approximately four weeks after the experiment began (i.e. end of hatching phase), larvae were being housed in 48 containers (from prolarvae to third instar; 12 females × 4 treatments).

At this point, third or fourth-instar larvae were individually housed in separate transparent plastic containers (50 mL, 2.5 cm diameter, 11 cm height) to avoid cannibalism. Housing and treatment conditions were the same as before separation. Over 200 larvae were tested, with at least 50 per treatment and at least 5 originating from the same mother. Remaining larvae were kept as reserves in the original containers and under the same regime; dead experimental larvae were replaced with an individual from the same treatment and clutch (when possible). Upon developing to the sixth instar, larvae were fed a live *T*. *tubifex*. Throughout the experiment, equal amounts of food were supplied to each container. After the sixth instar, larvae were reared until death or the (pen)ultimate instar; each subject was then preserved in 70% ethanol and measured (see the following section). The induction experiment was concluded by the end of May 2016.

### Measurements of larvae

**Fig 1:**
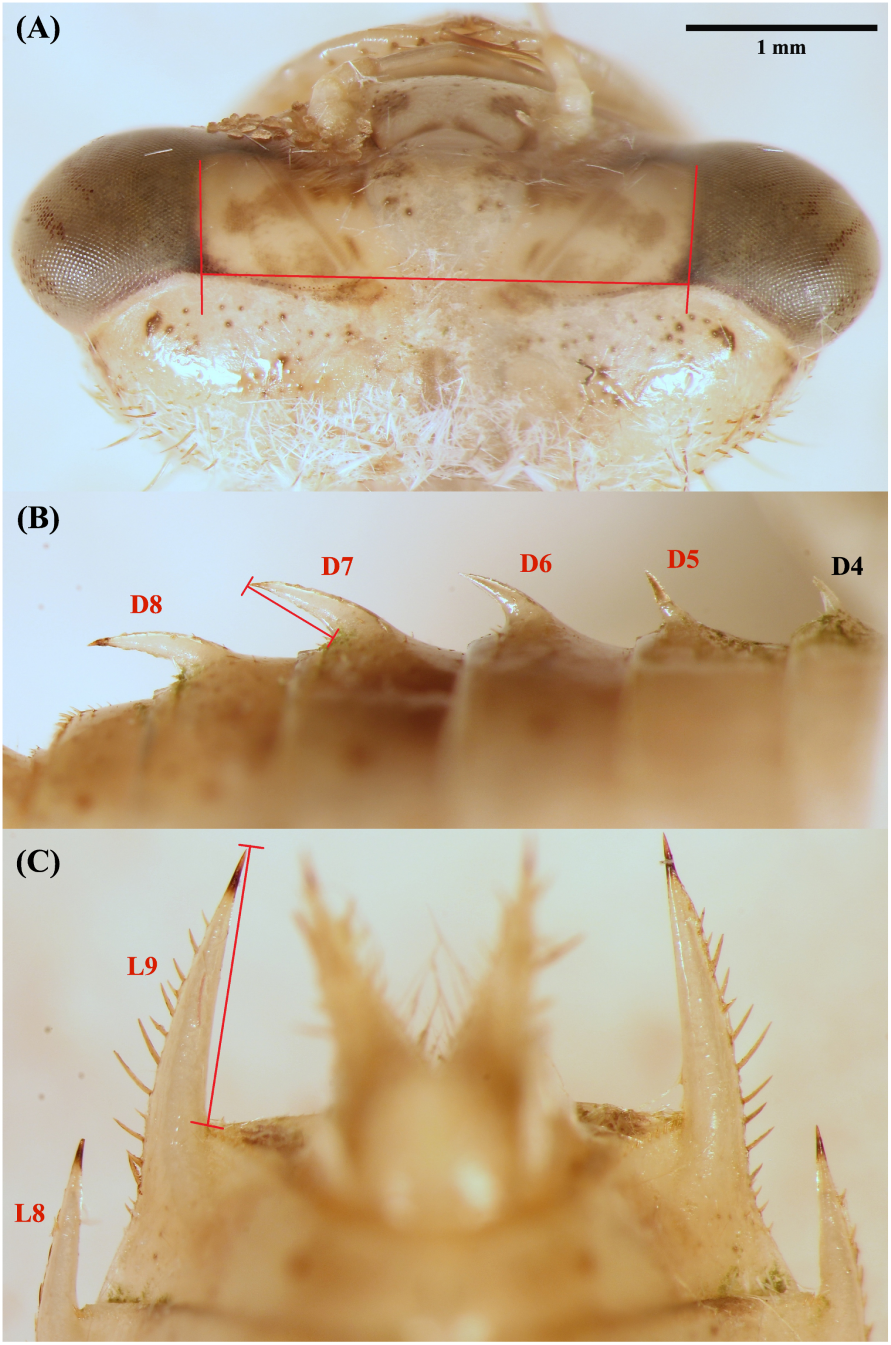
Measurements of larval *Sympetrum depressiusculum* after induction experiment. **(A)** head width; **(B)** dorsal and **(C)** lateral spines were measured from the base of each segment to the tip of the spine along the interior margin of the spine.

Larvae were photographed using an Olympus SZX7 stereomicroscope (Olympus Corporation, Japan) equipped with Canon 1100D camera (Canon U.S.A., Inc, United States). Three photos were taken per larva. The first was a dorsal view of the head for measuring head width, the most reliable measure of body size in dragonfly larvae [63]. The second was a dorsal view of lateral spines, while the third was a side view with dorsal spines visible. Measurements were performed using Quick Photo Camera 2.3 software (PROMICRA, s.r.o., Czech Republic). We measured spines known to act as a defence against fish predators (e.g. [35–37]). These included two lateral spines on abdominal segments VIII and IX (hereafter L8 and L9, respectively), as well as four dorsal spines on abdominal segments V, VI, VII, and VIII (hereafter D5–D8, respectively). The dorsal spine on segment IV was not measured because its small size and hidden position below the wing pads in (pen)ultimate-instar larvae suggest minimal impact on survival and therefore low selective pressure (see [41]). Spine length was the shortest distance from the tip to base [35,39], and head width was the shortest distance between internal eye margins (for details, see Fig 1). Larvae were discarded if they originated from females that were not represented in every treatment. To increase accuracy, only larvae with head widths >2000 μm were measured for spine length, resulting in 186 larvae.

### Data analysis

All analyses were performed in R 3.4.1 [64]. First, we performed multivariate analyses to evaluate the pattern of the lengths of all spines among individual treatments. For this, the spine length was expressed as a ratio of head width to control for size differences among individual larvae [39]. Furthermore, we computed a Euclidean distance matrix using measurements for all six spines. Multivariate homogeneity of variance across treatments (fish, invertebrate, mixed, control) was tested using PERMDISP2, a multivariate analogue of Levene’s test [65], implemented with *betadisper* in the vegan package [66]. Next, using the same distance matrix, we performed a permutational multivariate analysis of variance (PERMANOVA, *adonis* function in vegan; [66]) to evaluate combined dissimilarity across treatments in the lengths of all six spines. Significance was assessed via the permutation test (999 permutations) with pseudo-F ratios, adding a constraint for the originating female to eliminate possible maternal effects. Results of the PERMANOVA were visualized as a principal coordinates analysis (PCoA) plot in Canoco 5 [67]. For multivariate analyses, we used only larvae with data for all six spines (131 larvae, see S1 Appendix).

Second, six separate linear mixed-effect models (LMMs, *lme* in the nlme package; [68]) were used to test the fixed effect of treatment (explanatory variable) on the length of individual spines (dependent variables, L9, L8, D8–D5). Maternal influence on spine length was controlled for via setting originating female as a random effect. Similarly, head width was set as a random effect to control for size differences among individual larvae [39]. The significance of individual predator treatments (fish, invertebrate, mixed) was tested against the control treatment using the generic function *summary*, producing result summaries of our linear mixed-effect models. Each LMM for spines L9, L8, D8, D7, D6, and D5 used 182, 184, 184, 179, 172, and 147 measurements, respectively (see S1 Appendix).

## Results

**Fig 2:**
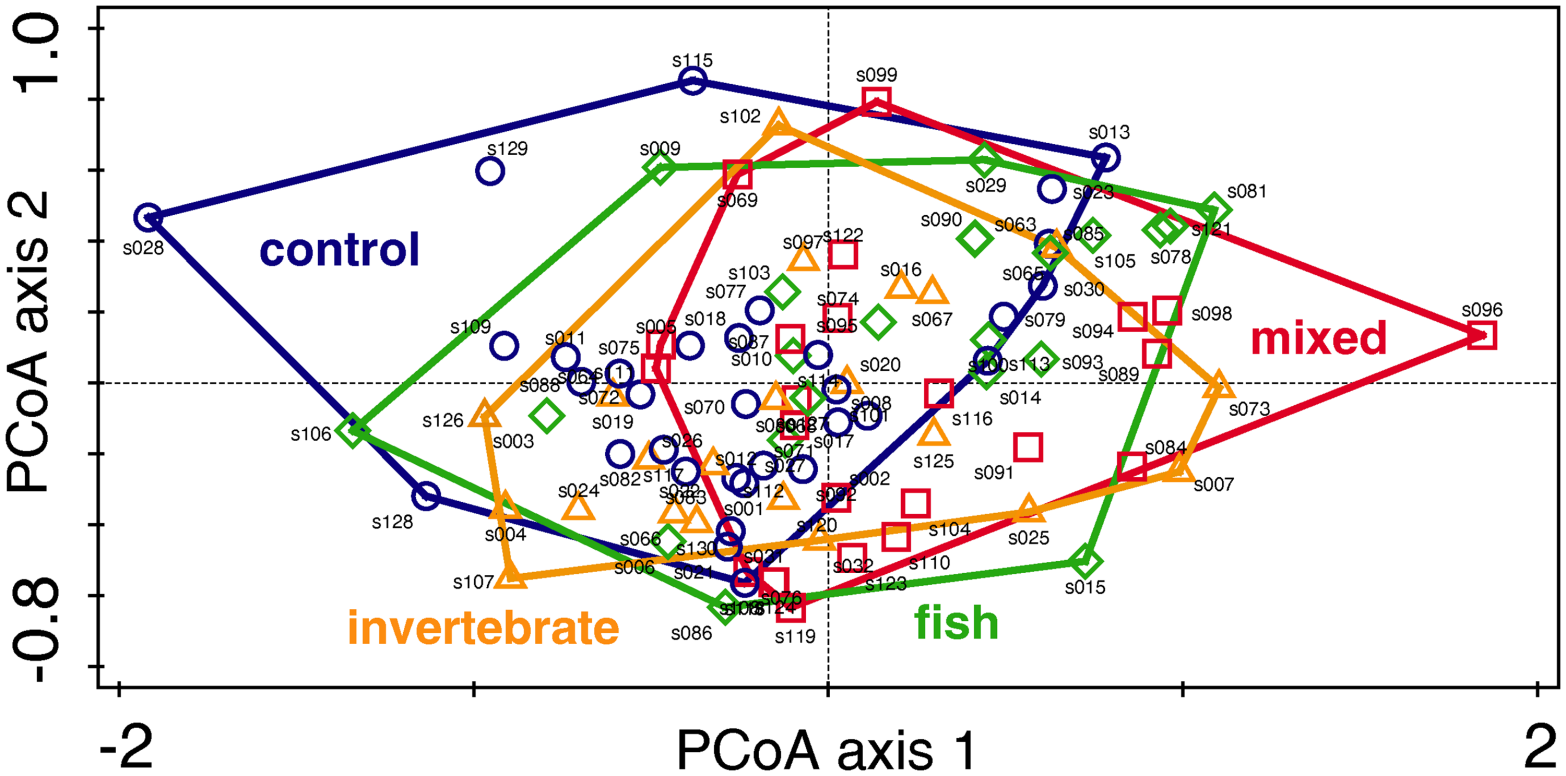
Principal coordinates analysis (PCoA) plot showing significant dissimilarities (*P* = 0.027) between predator treatments (fish, invertebrate, mixed, control) in spine length. Each point represents the position of an individual larva in the ordination space based on the measurements of all six spines. Length is expressed as spine length/head width.

**Fig 3:**
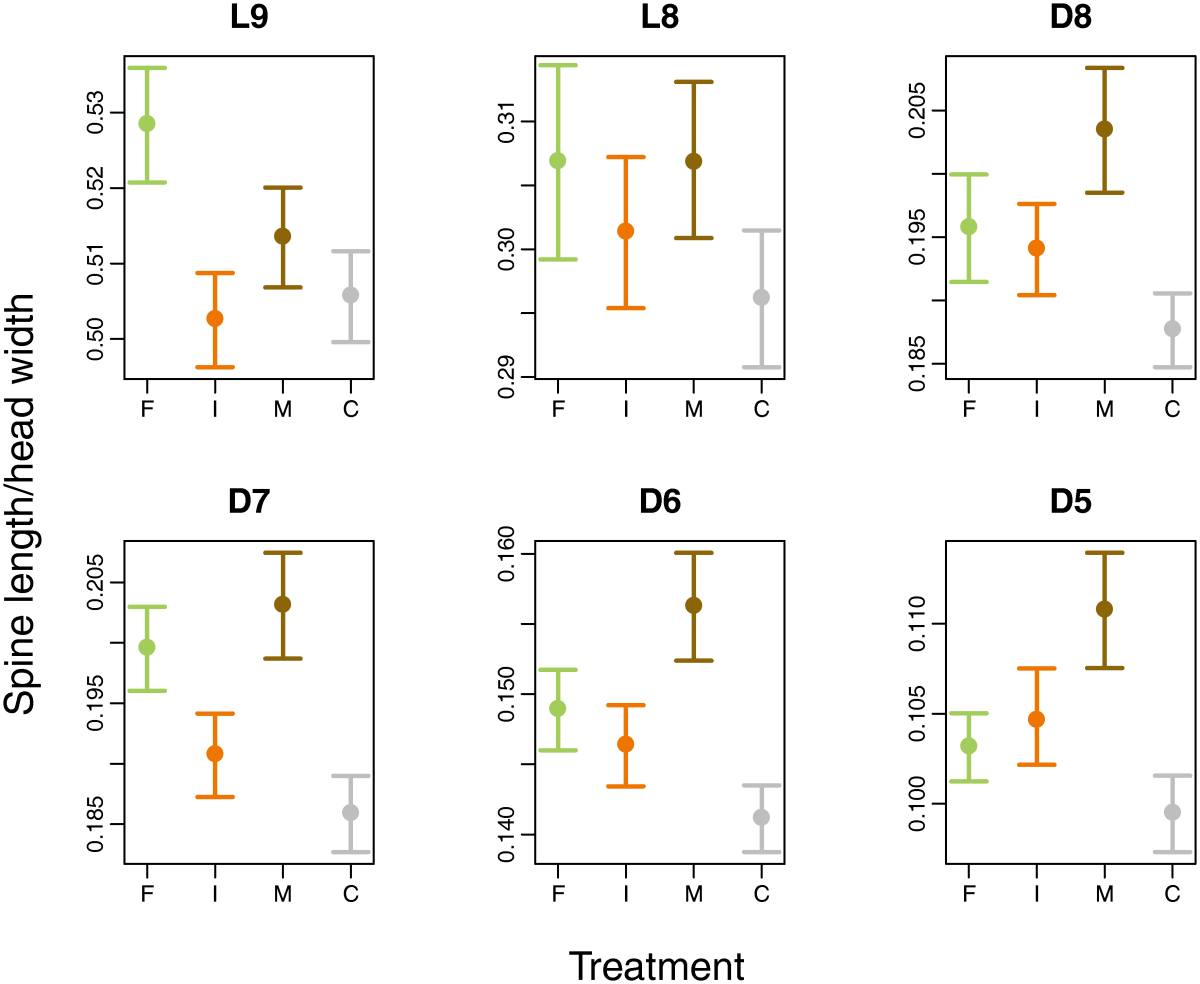
Line plots showing differences in mean spine lengths (L9, L8, D8–D5) across predator treatments (F = fish, I = invertebrate, M = mixed, C = control). Length is expressed as spine length/head width. Points and lines represent means and standard errors, respectively.

Group dispersion of the six spines was homogenous across treatments (PERMDISP2, minimal *P* = 0.866), but spine length significantly differed (PERMANOVA, df = 3, F = 2.72, *P* = 0.027; Fig 2). According to LMMs, fish cues resulted in longer L9 and D7 spines (i.e. one lateral, one dorsal) compared with the control, while mixed cues increased the lengths of D8–D5. Spine lengths in the invertebrate treatment did not differ from the control (Table 1, Fig 3).

**Table 1:** Test statistics from linear mixed-effect models (LMMs) showing spine length differences across predator treatments (fish, invertebrate, mixed) compared with control.

		*L9*	*L8*	*D8*	*D7*	*D6*	*D5*
*fish*							
	df	169	171	171	166	159	134
	t-value	1.994	1.305	1.497	2.784	1.844	1.114
	P-value	0.048*	0.194	0.136	0.006*	0.067	0.267
*invertebrate*							
	df	169	171	171	166	159	134
	t-value	-0.614	0.486	1.310	0.939	1.071	1.804
	P-value	0.540	0.628	0.192	0.349	0.286	0.073
*mixed*							
	df	169	171	171	166	159	134
	t-value	-0.681	0.078	2.013	2.328	2.600	2.547
	P-value	0.497	0.938	0.046*	0.021*	0.010*	0.012*

A separate LMM was performed per spine (L9, L8, D8–D5).

* indicates significance at P < 0.05.

df = degrees of freedom.

## Discussion

Contrary to our first hypothesis [41,42], our induction experiment provided evidence of spine-length plasticity in a long-spined, fish-habitat specialist. In accordance with our second hypothesis, *S*. *depressiusculum* larvae responded to fish chemical cues, increasing the lengths of defensively important spines (L9, D7) [36,39]. This result is generally congruent with the limited laboratory studies on fish cue-induced changes in spine length among medium-spined dragonfly generalists [38,39,46]. Although comparatively fewer spines responded significantly to fish cues, the observed changes nevertheless demonstrate that even specialists can dynamically increase defensive efficiency under actual predator presence.

Interestingly, *S*. *depressiusculum* larvae in the fish-cue treatment responded like *L*. *intacta* exposed to predator-borne (odour, diet) and prey-borne (alarm) cues combined (for details on terminology see [15,39]). Hence, larval *S*. *depressiusculum* altered their morphology even without diet and alarm cues that provide direct information about predation risk, suggesting that phenotypic plasticity in dragonflies is triggered primarily through predator-specific cues. This conclusion corresponds to previous research supporting the induction of behavioural responses through pre-consumption prey-borne cues, whereas morphological changes rely primarily on predator-borne cues (reviewed in [14,20]). However, both predator odour and diet cues may be needed for some species to exhibit full antipredator response (e.g. [69–71]). More experiments with different cue combinations are recommended to fully clarify phenotypic plasticity in dragonflies, for instance how larval morphology would change under exposure to both predator- and prey-borne cues. It was beyond the scope of our current study to include treatments containing diet-mediated and alarm cues. Furthermore, such manipulations would require hundreds of *S*. *depressiusculum* to serve as prey, which would be an inappropriate experiment given the fact that, according to the most recent study [53], the species is declining throughout Europe and may now be endangered. A new IUCN assessment should be undertaken to determine the current status of the species. However, similar studies can be undertaken with species that are more widespread.

In accordance with our second hypothesis, invertebrate predator cues did not elicit a morphological response significantly differing from the control. This result suggests that plasticity in these dragonflies is a strategy directed specifically against a single predator type and is unresponsive to others. A similar pattern was found in specialized freshwater snails [72], implying that targeted plasticity may be common among specialists. As a result, shifts in the dominant predator (i.e. from fish to invertebrate) will increase risk for *S*. *depressiusculum*. The potential danger may explain why these long-spined dragonflies retain phenotypic plasticity in response to fish predators; if spine length is kept at minimum when fish are absent, then shorter spines may be beneficial if an invertebrate predator appears in the environment. Spineless or reduced-spine species specialized to fishless habitats (e.g. *Sympetrum danae* or *S*. *fonscolombii*) may also employ a similar plasticity strategy. Indeed, field surveys confirmed limited plasticity to fish cues in spineless *Leucorrhinia glacialis* [41]. In contrast, broadly distributed generalists (i.e. species with intermediate spine length such as *Sympetrum sanguineum* and *S*. *vulgatum*) may also be generalist in their phenotypic plasticity. Future studies should examine whether such generalists respond to both fish and invertebrate predator cues. Overall, the degree of plasticity exhibited by specialists will still result in disadvantages for long-spined and short-spined species in invertebrate-heavy and fish-heavy environments, respectively. Even with inducible defences, however, generalists will suffer greater mortality than an appropriate specialist in its optimal environment, a common trade-off associated with the generalist-specialist spectrum [73].

Because we did not expect a reaction to invertebrate cues, we hypothesized that larvae reared under the mixed treatment would develop intermediate spines from a dosage effect. Instead, strikingly, all measured dorsal spines (D8–D5) were longer in these larvae than in fish-cue larvae (Fig 2); lateral spines showed no response. Previous studies in environments with multiple predators inducing conflicting phenotypes are biased toward behavioural responses and limited in taxonomic coverage. However, they suggest either an intermediate phenotype that balances overall predation risk [74] or a phenotype biased towards the more dangerous predator [71,75–79]. In general, the result of selective pressure from multiple predators will depend on the relative benefits of opposing phenotypes, and on any energetic or developmental constraints [79]. Although trade-offs in energy allocation are a major underlying assumption of phenotypic plasticity [16,80], data on development time and final-instar size in dragonflies do not support the hypothesis that expressing elongated spines requires additional costs [46], unlike other freshwater taxa (e.g. [32,78]). Additionally, developing longer spines in response to fish may reduce exocuticle thickness, but this change does not necessarily decrease protection (for details see [40]). To our knowledge, no previous study has reported the development of a phenotype that was more extreme under combined predator exposure than under single predator exposure. The results of our mixed treatment suggest that *S*. *depressiusculum* larvae can detect invertebrate predators, but their morphology cannot respond solely to such predators.

Thus, larval *S*. *depressiusculum* probably perceive an environment with multiple predators as riskier than a single-predator environment. In our study, predator biomass in the treatments was not equal. However, when predators tend to induce opposing phenotypes, the amount of cue from each predator is not a critical factor [71]. Moreover, evidence from other freshwater taxa suggests that inducible defences often become saturated at relatively low predator densities; in such cases, increasing predator density does not elicit a more extreme prey phenotype (e.g. [20,23,71,78,81]). In our experiment, predator densities (i.e. concentration of predator cues) were very high (c.f. [35,38,39,42]). We thus expected a maximum saturation of predator cues in each treatment. Since the mixed treatment basically contained half the concentration of cues per predator type, we propose that the observed exaggerated morphology can be related to behavioural antipredator strategies rather than simply altered cue concentrations. Indeed, organisms show both morphological and behavioural adaptations to avoid predation [82–84]. In dragonflies, larvae use jet propulsion to burst-swim away from both fish and invertebrate predators [36,42]. Evidence from the *Leucorrhinia* system suggests that a fish-habitat specialist like *S*. *depressiusculum* exhibits high burst-swimming speed [44,45]. While elongated dorsal spines increase handling time [35] and often lead to rejection from the fish predator [36,37], shorter lateral spines are likely a major advantage when burst-swimming away from an aeshnid predator. Therefore, in *S*. *depressiusculum*, extremely long dorsal spines may offset short lateral spines when facing fish predators, whereas short lateral spines can facilitate a rapid escape from ambush invertebrate predators, mitigating the presence of elongated dorsal spines.

In natural communities, fish are also likely to consume aeshnid predators, thereby causing the insects to alter their behaviour and avoid areas with fish (density- and trait-mediated indirect interactions [78]). As a result, fish will reduce the encounter rates of larval *S*. *depressiusculum* with invertebrate predators. Indeed, studies from the *Leucorrhinia* system typically report either fish-dominated or aeshnid-dominated habitats, not both (e.g. [42]). Therefore, our experiments simulated conditions that may rarely occur in nature. Nonetheless, our results suggest that the unidirectional spine-elongation response of *S*. *depressiusculum* may be an efficient strategy against both types of top predators, suggesting that some selective pressure from combined predators does exist. We recommend additional studies that could clarify whether the presence of multiple predators is more common than previously supposed. Such new data would lend ecological validity to the data presented here and provide a basis for behavioural studies on fish-habitat specialists facing multiple predators.

The evolution of fixed or plastic defensive strategies depends on the frequency of experiencing contrasting environments, along with the costs and benefits of alternative phenotypes [22,85]. Interestingly, our study shows that fish-habitat specialists may favour phenotypic plasticity over constitutive defence, even when they rarely encounter a habitat with alternative predators. This outcome may be because a constitutive defence trait has not yet been fixed in this population [72]. However, phenotypic plasticity in *S*. *depressiusculum* larvae may be an effective strategy against fish predators, while also acting as insurance against the possibility of encountering both fish and invertebrate predators in their lifetime. In our experiment, eggs were collected from adults captured at a site containing high densities of phytophagous cyprinid fish and aeshnid predators. Studies on fish-borne cues that induce defences suggest conservation of the chemical eliciting plastic responses, even across different fish families and feeding strategies (e.g. [19]). Therefore, larvae may morphologically react to phytophagous fish that do not actually pose any threat to them, while also being under intense selective pressure from invertebrate predators. In other words, local adaptation of reaction norms could have affected the expression of plasticity in our experiment (cf. [39]). Nevertheless, our study shows that phenotypic plasticity is not specific to habitat generalists. Moreover, it suggests that dragonflies may be able to fine-tune their phenotypes to predator-competitor combinations, as found in other freshwater organisms [86]. In Central and Western Europe, *S*. *depressiusculum* populations are scattered, isolated, and continuously declining [52,53]. Our results indicate that its occurrence may be constrained by unsuitable abiotic conditions associated with the loss of natural habitats rather than predation.

## Supporting information

S1 Appendix Dataset of 186 larvae measured in an experiment focused on phenotypic plasticity in the spine length of larval *Sympetrum depressiusculum* (Odonata: Libellulidae). (XLSX)
